# Social support and C-reactive protein in a Québec population cohort of children and adolescents

**DOI:** 10.1371/journal.pone.0268210

**Published:** 2022-06-22

**Authors:** Eloïse J. Fairbank, Jennifer J. McGrath, Mélanie Henderson, Jennifer O’Loughlin, Gilles Paradis

**Affiliations:** 1 Department of Psychology, Concordia University, Montréal, Québec, Canada; 2 Department of Pediatrics, Université de Montréal, Montréal, Québec, Canada; 3 Centre de Recherche CHU Sainte-Justine, Montréal, Québec, Canada; 4 School of Public Health, Department of Social and Preventive Medicine, Université de Montréal, Montréal, Québec, Canada; 5 Department of Epidemiology, Biostatistics, and Occupational Health, McGill University, Montréal, Québec, Canada; Sun Yat-sen University, CHINA

## Abstract

**Objective:**

Robust evidence exists for the health-enhancing benefits of social support in adults. Inflammatory processes are thought to be an important mechanism linking social support and health risk. Less is known about the relation between social support and chronic inflammation during childhood and adolescence, or when the association emerges during the lifespan.

**Method:**

Data from the population-representative 1999 Quebec Child and Adolescent Health and Social (QCAHS) survey were analyzed. Youth aged 9, 13, and 16 years (*N* = 3613) and their parents answered questions about social support. A subsample (*n* = 2186) completed a fasting blood draw that was assayed for C-reactive protein (CRP).

**Findings:**

Higher social support was significantly associated with lower hs-CRP_log_, after controlling for age, sex, body mass index (BMI Z-score), medication use, puberty, ethnoracial status (French-Canadian), smoking, household income, and parental education (*F* = 25.88, *p* = < .001, Total *R*^2^_adj_ = 10.2%). The association was largely similar for boys and girls, and strengthened with age.

**Conclusion:**

Greater social support was linked to lower chronic low-grade inflammation in a large sample of children and adolescents. Effect sizes were small and consistent with prior findings in the adult literature. Importantly, these findings provide evidence that the relation between social support and inflammation emerges early in the lifespan. Future work should consider broader, more encompassing conceptualizations of social support, the role of social media, and prospective trajectories of social support and inflammatory markers.

## Introduction

Chronic inflammation is a key contributor to the pathophysiology of multiple health outcomes [1]. Chronic low-grade inflammation is characterized by increased levels of cytokines that are the long-term response to threats overtime (e.g., stressor exposure, injury), and result in elevated susceptibility to disease [2]. Proinflammatory cytokines include interleukin (IL)-1, IL-6, C-reactive protein (CRP), and tumor necrosis factor-alpha (TNF-alpha) [2]. CRP is an acute phase reactant secreted in response to both acute and chronic inflammation. Although CRP initially works to restore the body after infection or injury, high circulating levels of CRP have been linked to adverse health outcomes [3]. In fact, higher levels of proinflammatory circulating markers have been associated with osteoporosis, certain cancers, cardiovascular disorders, type 2 diabetes, rheumatoid arthritis, Alzheimer’s disease, frailty and disability, and increased risk of all-cause of mortality [4, 5].

Social support is conceptualized as the perception that “one is valued, cared for, and loved by others in a social network” [6]. Social support encompasses numerous experiences within one’s network, both negative and positive [7], and has important links to health outcomes [8–14]. In adults, social support is often parsed into types: *structural support* is characterized by one’s integration within their network; *functional support* is characterized by the kind of support (e.g., emotional), and can be further distinguished as *perceived* or *received* support [15]. In children, however, social support is traditionally parsed by the source or the particular people within the child’s social network: parents, teachers, peers, siblings, others (e.g., relatives, neighbors, coaches). Studies in the child and adolescent literature predominantly present findings based on only one source (e.g., parents, peers) [16]. While children’s positive and negative social relationships are commonly measured using this singular source approach, this introduces an important construct gap in the field because focusing narrowly on a single source (e.g., parents) fails to capture having *anyone* within one’s social network who can contribute to feeling valued and supported. Interestingly, within the adult literature, studies using a broader conceptualization of social support (e.g., aggregated measures across sources and types of support) observed stronger associations with health [9, 10]. Ultimately, the perceived cumulative support across one’s entire social network provides a comprehensive representation of social support.

### Social support, health, and inflammation

Social support is a robust predictor of health and well-being [8–14]. Indeed, social support has been found to be protective against cardiovascular disease, cancer, infectious disease, depression, and early mortality [14]. A seminal meta-analysis of over 300,000 adults indicated stronger social support was linked to 50% greater odds of survival, regardless of age, sex, initial health status, cause of death, or length of follow-up period [9]. In a study of over 100 countries, the association between social support and self-reported health was consistent across the socioeconomic gradient and geographic locations [10]. Inflammation, specifically proinflammatory cytokines (IL-6, CRP), has been found to be an important mechanism linking social support and health risk [17].

Social support is linked to inflammation through complex bidirectional processes. Two theories posit neurophysiological and behavioural pathways to explain these associations between social support and inflammation. The *social signal transduction theory* suggests that a lack of social support and/or interpersonal stress activate brain regions associated with cytokine release, which become repeatedly sensitized over time [18]. The *sickness behavior theory* postulates that sickness and resulting chronic cytokine exposure lead to altered social support (e.g., withdrawal, energy conservation, anxiety, hypervigilance) [19]. In other words, being sick may change how one interacts with others; they may reach out for support *or* isolate and stay home. Various studies also have considered plausible pathways that mediate the relation between social support and inflammatory processes, such as neurological, behavioural, psychological, and physiological mechanisms [20–23]. For example, animal models establish credibility of physiological causality linking social support and inflammation (e.g., unstable social environment and higher CRP in rabbits) [24]. Further, findings of an association between adverse social experiences and inflammation during adolescence provides preliminary evidence that cumulative exposure to strained or limited social support over the lifetime may not be necessary [25, 26]. The underlying pathophysiological mechanisms remain unclear and necessitate comprehensive approaches to disentangle the complex relations (and plausibly bi-directional relations) among social support, immune functioning, and inflammation.

### Social support and inflammation: Adulthood

Among adults, evidence consistently links social support to lower levels of chronic low-grade inflammation. Insufficient social support during adulthood (i.e., social isolation) increases inflammatory responses while suppressing antiviral immunity, whereas positive social support (i.e., integration) decreases inflammation and strengthens antiviral responses [27]. For example, a population-based study in the United States found that higher levels of social support and lower levels of social conflict within one’s social network predicted lower levels of inflammatory markers throughout adulthood [25]. In a recent meta-analysis of 47 studies, social support was significantly related to lower levels of inflammatory markers (*r* = -.073) [17]. Specifically, social support (e.g., family, caregiver, friends, neighbours, supervisors/co-workers) was negatively associated with inflammatory markers (i.e., CRP) in clinical and non-clinical samples. Given the robust association of higher social support and lower inflammation in adults (albeit of a small magnitude), it is important to examine if and when this association emerges during childhood and adolescence [28].

### Social support and inflammation: Childhood and adolescence

Compared to adulthood, less is known about the relation between social support and chronic inflammation during childhood and adolescence. Mounting evidence suggests that the association between social support and inflammation begins early in the lifespan [29–32]. Social support during childhood and adolescence (e.g., greater parent support, warmer family climate, lower interpersonal stress) has been linked to CRP [33–37], IL-4 [38], IL-6 production [39], and the inflammatory response [40]. For example, greater parental support is associated with lower levels of IL-4 [38], and has been found to moderate the association between CRP with depressive symptoms [33] and sympathetic activity [34] in adolescents. Warmer family climate (i.e., more emotional support, less conflict, less harsh) predicted IL-6 production trajectories over 18 months; and, having a less harsh family climate was a buffer when experiencing a major life event, leading to lower IL-6 production [39]. Curiously, although school environment (e.g., teachers, school connectedness) has been related to positive mental health outcomes and reduced health risk behaviours [41, 42], no studies to date have explored its relation to chronic inflammation. Strained social relationships, conversely related to emotional social support and warmth, have also been examined. For example, adolescents who reported more interpersonal stress across sources (i.e., peers, family, school) had higher levels of CRP (β = .28) [37]. Poor parental monitoring (e.g., lack of time or interest in teen’s activity) and negative parental behaviours (e.g., conflictual aggression) have been associated with higher CRP during adolescence [35, 36]. Additionally, fewer warm, supportive peer relationships predicted greater pro- and anti-inflammatory responses in adolescent girls six months later [40]. Less supportive home and neighborhood environments have also been associated with higher levels of CRP in children and adolescents [43, 44]. Together, these studies evidence modest associations between social support and inflammatory markers and suggest that this relation emerges early during development; however, the majority of studies were conducted with adolescents and largely focused on a singular source of social support.

### Rationale and objectives

The importance of understanding the pathophysiology of inflammation from a young age is timely and has lasting implications for overall health. Childhood and adolescence are important periods for developing interpersonal skills and lifestyle behaviors. Inadequate social support during childhood (e.g., social isolation) is linked to higher levels of inflammation in adulthood nearly 40 years later [29–32]. Current findings suggest the association between greater social support and lower chronic inflammation, or alternatively more strained relationships and higher chronic inflammation, may emerge during adolescence. However, most studies conducted to date have been conducted in small samples (e.g., *N* < 200) and almost exclusively use narrow social support measures based on certain sources (e.g., parents, peers). Further, to the best of our knowledge, no studies have considered sex differences. Therefore, before we can advance our understanding of the pathophysiological mechanisms underlying the association between social support and inflammation, it is necessary to first establish whether the association exists at different ages across childhood and adolescence using large, population cohorts.

The aim of the present analysis was to test the association between social support and chronic low-grade inflammation (CRP) in a population-based sample of children and adolescents. To address the existing gaps within this literature, the specific objectives were to i) replicate previous findings among adults and adolescents that observed greater overall social support was associated with lower CRP; ii) extend previous work by examining the association during childhood; and, iii) examine this association within a large population sample. As a secondary aim, potential sex- and age-based differences were explored to test whether the relation was similar in boys and girls, and across 9-, 13-, and 16-year-olds.

## Methods

### Population dataset

The Quebec Child and Adolescent Health and Social Survey (QCAHS) was a multi-stage sample survey of Quebec youth aged 9 years (*n* = 1267), 13 years (*n* = 1186), and 16 years (*n* = 1160; Total *N* = 3613) that was population-representative at the time of sampling (1999). Complete survey design and methodology are reported elsewhere [45, 46]. The original QCAHS survey was approved by the Ethics Review Board of Direction Santé Québec, Institut de la Statistique du Québec, and CHU Sainte-Justine; secondary dataset use was approved by Concordia University’s Ethics Committee (UH2006-068).

### Protocol

Research teams completed standardized assessments during morning sessions at schools of blood pressure, anthropometrics (height, weight, waist circumference), and >10hr fasting blood draw. Parents and youth completed questionnaires of items previously validated and adapted from other Quebec or Canada population-based surveys (e.g., Canadian National Longitudinal Study of Children and Youth; Québec Enquête Sociale et de Santé). Questionnaires were administered in the language of instruction at the child’s school (e.g., French or English) [46].

### Measures

#### C-reactive protein (CRP)

Blood was obtained by venipuncture by a pediatric nurse and high-sensitivity C-reactive protein (CRP) concentrations were assessed with the IMMAGE^®^ immunochemistry system (Beckman Coulter), with a lower detection limit of 0.20 mg/L per assay [47]. Assay values below the lower detection limit were imputed using multiple imputation, which is deemed preferable to assigning a static value of 0.20 mg/L [48]. CRP concentrations were log^e^-transformed to adjust for non-normality and positive skew [49]. Over half of the sample agreed to have blood drawn (*N* = 2475; age 9, 62%, *n* = 783; age 13, 69%, *n* = 818; age 16, 75%, *n* = 874). The present analyses include youth who completed the blood draw, consented to C-Reactive Protein assays, had sufficient plasma volumes, and had no chronic health condition known to affect CRP (e.g., diabetes, cystic fibrosis, inflammatory bowel disease; *N* = 2232; age 9, *n* = 710; age 13, *n* = 715; age 16, *n* = 807). CRP concentrations are remarkably stable over time with no seasonal variations and only occasional spikes due to acute infections; the consistency of measures repeated years apart is comparable to cholesterol (i.e., *r* = 0.50) [50, 51]. Previous QCAHS analyses examined non-response and/or selection bias; there were no statistically significant differences for blood draw completion for sex, pubertal status, smoking, weight status, parental smoking, parent education, household income, or school setting (rural or urban) [46]. Language spoken at home (age 9 only; 67% francophone, 53% anglophone) and physical activity levels (age 16 only; 72% active, 81% least active) were significantly different between those who did and did not complete the blood draw [46]. To address possible bias within the CRP subsample used in the present analyses, sample demographics were compared to the entire QCAHS sample. Effect sizes were estimated for continuous and categorical variables (Cohen’s *d* and ϕ Phi coefficient).

#### Social support

Youth and parents answered items about social support. QCAHS questionnaires included social support items drawn from surveys previously used in Quebec population surveys (e.g., Styles de vie des jeunes du secondaire en Outaouais study [52]; Ado, Familles et Milieu de vie study [53]) and some items adapted from the Social Support Rating Scale [16]. A panel of independent raters (*n* = 3) reviewed child and parent questionnaires to identify potential items that captured social support broadly (e.g., confiding, perceived, emotional, warmth) with any source (e.g., parents, siblings, peers, teachers, neighbors, relatives, friends). Raters had excellent agreement (kappa (κ) = .983, *p* < .001): 38 items were identified for children (age 9); 44 items were identified for adolescents (ages 13, 16: same 38 items, plus 6 additional items not answered by children). Examples of items included “*Do you think your [friend] would really listen to you and help you feel better if you really needed it*?”, “*Do your neighbors help each other*”, and “*Do some of your teachers listen to you when you need to talk about your problems*”. Items were rated on Likert scales (e.g., *Completely disagree* to *Completely agree*, -2 to 2; *Never to Very often*, 0 to 3) and harmonized to yield consistent directions (i.e., higher score = more social support). A cumulative social support score was derived across items; summed scores could range from -60 to 60. In the present sample, the social support score evidenced good internal consistency (Cronbach’s α = .817).

#### Covariates

Youth reported their age and sex (response options only included boy or girl; insufficient information to discern biological *sex* assigned at birth versus identified *gender*. Sex is the assumed construct measured, consistent with prior QCAHS publications). Age was verified using Québec Ministry of Education records, which were based on birth certificate information. Height and weight were recorded based on standardized protocols (see [46]). Body Mass Index (BMI) was calculated [weight (kg)/height^2^(m)] and converted into age- and sex-specific Z-scores based on the Centers for Disease Control and Prevention (CDC) standardized curves [54]. Pubertal status was defined as adrenarche stage (i.e., body hair, pubic, underarm). Smoking status was defined as smoking a cigarette ever (age 9) or over past 30 days (age 13 and 16; 1994 Canadian Youth Smoking Survey [55]. Medication use was operationally defined as use of any over-the-counter medications or prescription drugs for possible infection or inflammation in the prior 2 weeks (e.g., antibiotics, pain/fever, cold/allergies, respiratory problems, pump/inhaler) [47]. Socioeconomic status (SES) was defined as parental education (no formal schooling to university) and total household income (1998, before taxes and deductions; <$10K to ≥$80K CAN) based on parent report. Ethnoracial background was defined by categorizing children as French-Canadian (i.e., parents born in Quebec or Canada, and French language spoken at home) or not. This variable has been used in prior QCAHS publications as a proxy for ethnoracial status (e.g., ethnicity, race), which was unavailable.

#### Data integrity and missingness

Assumptions of linearity, normality, and residuals (independent, normality with mean of zero, homogeneity) were verified [49]. Univariate outliers were retained when clinically plausible (e.g., BMI-Z score). Sensitivity analyses were conducted with and without CRP values exceeding 10 mg/L (*n* = 45), which is the designated clinical threshold suggesting active infection. Data were missing completely at random (MCAR *χ*^*2*^ = 17.830, *df* = 18, *p* = .467; 12.2% total missingness). Multiple imputation with fully conditional specification (iterative Markov Chain Monte Carlo, MCMC) was used to impute 25 datasets that were inspected for multivariate outliers (*n* = 4). Sensitivity analyses were also conducted using imputed and non-imputed datasets. Results were largely identical; therefore, only imputed results are presented for parsimony. Analyses were performed using SPSS version 27.

#### Hypothesis testing

Linear regression using the General Linear Model (GLM) was used to test the hypothesized relation that greater social support would be associated with lower CRP. GLM is deemed preferential to TOBIT models (e.g., censored regression) when combined with multiple imputation to address data missing below-detectable-limits (e.g., non-detects) [48]. Prediction models adjusted for covariates (age, sex, BMI Z-score, medication use, puberty, smoking status, French-Canadian status, household income, parental education). Models were also stratified by sex (boy, girl) and by age (9, 13, 16 years) for secondary analyses to explore results and inform the interpretation. Effect sizes were estimated for models and covariates (R^2^ and η^2^_p_ eta-squared). Finally, sensitivity analyses were conducted to examine the effects of CRP values above the clinical threshold and imputed data.

## Results

### Sample demographics

The present analyses were based on the CRP subsample (*n* = 2186) who completed the blood draw and consented to CRP assaying, as described above. Demographic data are presented in Table 1. This subsample included a similar percentage of boys and girls (48.5%, 51.5%, respectively), who were of normal weight status (BMI-Z _avg_ = .203), predominantly non-smoking (80.8%), and lived in households with an average income of $48.25K CAN and with a parent who completed a university degree or higher (70.3%; ≥16 yrs schooling). Youth had low hs-CRP levels (0.831 mg/L); girls had higher levels of hs-CRP than boys (*t* = 3.713, *p* < .001; Cohen’s *d* = 0.159). Compared to the entire QCAHS sample, the CRP subsample was ~3 months older (*t* = 3.173, *p* = .002; Cohen’s *d* = 0.086) and had slightly more advanced pubertal development (0.08 adrenarche stage; t = 2.647, *p =* .008; Cohen’s *d* = 0.072); the observed effect sizes imply these differences were trivial. There were no differences for sex, BMI Z-score, current smoking status, medication use, household income, parental education, nor cumulative social support score.

**Table 1:** Sample demographics.

	QCAHS (*N* = 3613)				Present Analysis (*n* = 2186)				QCAHS vs. CRP
	Complete Sample	Boys	Girls	Sex Comparison	CRP Subsample	Boys	Girls	Sex Comparison	
	*M (SD)*	*M (SD)*	*M (SD)*		*M (SD)*	*M (SD)*	*M (SD)*		
**Age** ^a^	12.49 (2.90)	12.45 (2.88)	12.53 (2.92)	*t* = .829,	12.74 (2.91)	12.69 (2.87)	12.79 (2.94)	*t* = .804,	*t* = 3.173,
								*p* = .422	*p* = .002
				*p* = .407				*d* = .034	*d* = .086
				*d* = .028					
**BMI Z-Score** ^a^	.192 (1.08)	.233 (1.08)	.154 (1.09)	*t* = 2.181,	.203 (1.05)	.217 (1.06)	.189 (1.03)	*t* = -.626,	*t* = 0.379,
				*p* = .029				*p* = .531	*p* = .704
				*d* = .073				*d* = .027	*d* = .010
**Pubertal stage** ^a^	2.57 (1.11)	2.46 (1.04)	2.67 (1.16)	*t* = 5.646,	2.65(1.11)	2.48 (1.04)	2.76 (1.15)	*t* = 5.959,	*t* = 2.647,
									*p* = .008
				*p*<.001				*p*<.001	
									*d* = .072
				*d* = .191				*d* = .255	
**Income** ($K)^a^	48.29 (22.55)	48.33 (22.55)	48.25 (22.55)	*t* = .095,	48.25 (22.59)	48.53 (22.74)	47.98 (22.46)	*t* = -.569,	*t* = .062,
								*p* = 570	*p* = .950
				*p* = .924				*d* = .024	*d* = .002
				*d* = .004					
**Education** (years)^a^	14.13 (2.05)	14.13 (2.04)	14.13 (2.05)	*t* = .000	14.12 (2.04)	14.13 (2.03)	14.11 (2.04)	*t* = -.230,	*t* = .174,
				*p* = 1.000				*p* = .818	*p* = .862
				*d* = .000				*d* = .010	*d* = .005
**CRP** (mg/L)					0.831 (1.38)	.719 (1.26)	.937 (1.47)	*t* = 3.713,	
								*p* =<.001	
								*d* = 0.159	
**Social support** (cumulative score)	20.21 (10.28)	19.75 (10.05)	20.67 (10.48)	*t* = 2.692,	20.23 (10.43)	19.58 (10.55)	20.84 (11.01)	*t* = 2.729,	*t* = .071,
				*p* = .007				*p* = .006	*p* = .943
				*d* = .090				*d* = 0.118	*d* = .002
	***n* (%)**	***n* (%)**	***n* (%)**	**Sex Comparison**	***n* (%)**	***n* (%)**	***n* (%)**	**Sex Comparison**	
**Sex**		1778 (49.2)	1835 (50.8)			1061 (48.5)	1125 (51.5)		χ^2^ = .267,
									*p* = .605
									ϕ = .007
**Medication Use** (yes)	1655 (45.80)	729 (41.0)	926 (50.5)	χ^2^ = 32.569,	1046 (47.80)	457 (43.10)	589 (52.40)	χ^2^ = 18.856,	χ^2^ = 2.189,
				p<.001				*p*<.001	*p* = .139
				ϕ = .095				ϕ = .093	ϕ = .019
**French-Canadian** (yes)	2892 (80.0)	1455 (81.80)	1437 (78.30)	χ^2^ = 7.016,	1746 (79.90)	882 (83.1)	864 (76.8)	χ2 = 13.605,	χ^2^ = .008,
				p = .008				*p*<.001	*p* = .927
				ϕ = .044				ϕ = .079	ϕ = .001
**Smoking** (yes)	666 (18.40)	288 (16.2)	378 (20.6)	χ^2^ = 11.635,	419 (19.2)	174 (16.40)	245 (21.80)	χ2 = 10.194,	χ^2^ = .573,
				p = 0.001				*p* = .001	*p* = .449
				ϕ = .057				ϕ = .068	ϕ = .010

Note.

^a^Test statistics (t-tests) calculated on all available data from QCAHS (*n*<3613).

### Model testing

In the prediction model, known covariates (sex, age, BMI Z-score, pubertal status) were significantly associated with hs-CRP_log_ (see Table 2). Additional covariates were retained in the prediction models for consistency with past QCAHS publications and because they have been previously linked to CRP in other youth samples (e.g., medication use, French-Canadian status, smoking status, household income, parental education) [56, 57]. A higher cumulative social support score was significantly associated with lower hs-CRP_log_, after controlling for the aforementioned covariates. Social support accounted for 0.3% of the variance, yielding a small effect size, which is consistent with prior findings in the adult and adolescent literature. Overall, the prediction model accounted for 10.2% of the variance (see Table 2).

**Table 2:** Prediction model.

CRP Sample (*N* = 2186)					
	Unstd. *B*	Unstd. *SE*	*t*	*p*	η^2^_p_
	(F = 25.88, p < .001, Adjusted R^2^ = .102)				
**Sex** (ref: Boys)	0.138	0.032	4.345	< .001	.011
**Age**	0.047	0.010	4.547	< .001	.012
**BMI-Z Score**	0.177	0.014	12.507	< .001	.076
**Medication Use** (ref: No)	0.059	0.031	1.878	.061	.002
**Pubertal status**	-0.070	0.027	-2.578	.010	.004
**French-Canadian status** (ref: No)	0.028	0.041	0.670	.503	.000
**Smoking status** (ref: No)	-0.044	0.043	-1.006	.315	.001
**Household income**	0.000	0.001	-0.639	.523	.000
**Parental education**	-0.008	0.009	-0.843	.400	.001
**Social support** (cumulative score)	-0.311	0.150	-2.078	.038	.003

Secondary analyses stratified by sex and age yielded largely consistent associations (i.e., magnitude of effect). Analyses stratified by sex revealed that the findings were largely similar for boys and girls; however, these analyses failed to reach statistical significance (see Table 3). The slope and magnitude of the association between social support and hs-CRP_log_ were comparable in boys and girls (Unstd. *B* = -.325, η^2^ = .003; Unstd. *B* = -.304, η^2^ = .003; respectively).

**Table 3:** Prediction model by sex.

	Boys					Girls				
	Unstd. *B*	Unstd. *SE*	*t*	*p*	η^2^_p_	Unstd. *B*	Unstd. *SE*	*t*	*p*	η^2^_p_
	(F = 12.79, p = <.001, Adjusted R^2^ = .091)					(F = 15.28, p = <.001, Adjusted R^2^ = 0.103)				
**Age**	0.034	0.013	2.498	.013	.007	0.060	0.014	4.127	<.001	.019
**BMI-Z Score**	0.169	0.020	8.567	<.001	.073	0.185	0.020	9.257	<.001	.079
**Medication Use** (ref: No)	0.085	0.044	1.942	.052	.005	0.035	0.045	0.791	.429	.001
**Pubertal status**	-0.033	0.038	-0.864	.388	.001	-0.102	0.037	-2.780	.006	.009
**French-Canadian status** (ref: No)	0.029	0.058	0.495	.621	.000	0.022	0.054	0.413	.680	.000
**Smoking status** (ref: No)	-0.098	0.064	-1.526	.128	.003	-0.006	0.057	-0.113	.910	.000
**Household income**	-0.001	0.001	-0.845	.399	.001	0.000	0.001	-0.019	.985	.000
**Parental education**	0.009	0.013	0.724	.470	.001	-0.024	0.012	-1.992	.047	.005
**Social support** (cumulative score)	-0.325	0.221	-1.472	.142	.003	-0.304	0.207	-1.466	.143	.003

Analyses stratified by age revealed that the association between social support and hs-CRP_log_ increased in magnitude across ages (see Table 4). While the beta coefficients for cumulative social support score in 13- and 16-year-olds were similar in magnitude to those of the non-stratified prediction model, they did not reach significance (η^2^_p_ = .002 and .004, respectively). Specifically, beta coefficients increased in a gradient fashion with age (Unstd. *B =* .002, -.240, and -.367, respectively). Further, these similar effect sizes for social support were observed while controlling for other meaningful covariates, such as BMI Z-score. Finally, sensitivity analyses were conducted to examine the effects of including CRP values above the clinical threshold (>10) and using original (non-imputed) data; overall results were largely identical (not shown for parsimony).

**Table 4:** Prediction model by age.

	9-year-olds (*n* = 696)					13-year-olds (*n* = 704)					16-year-olds (*n* = 786)				
	Unstd. *B*	Unstd. *SE*	*t*	*p*	η^2^_p_	Unstd. *B*	Unstd. *SE*	*t*	*p*	η^2^_p_	Unstd. *B*	Unstd. *SE*	*t*	*p*	η^2^_p_
	(F = 8.44, p<.001, Adjusted R^2^ = .088)					(F = 11.81, p<.001, Adjusted R^2^ = .121)					(F = 8.44, p<.001, Adjusted R^2^ = .079)				
**Sex** (ref: Boys)	0.162	0.052	3.090	.002	.016	-0.043	0.059	-0.727	.468	.002	0.231	0.053	4.315	<.001	.027
**BMI-Z Score**	0.165	0.023	7.144	<.001	.080	0.219	0.025	8.818	.000	.118	0.156	0.026	5.886	<.001	.051
**Medication** (ref: No)	0.025	0.057	0.444	.657	.001	0.027	0.053	0.517	.606	.001	0.105	0.052	2.026	.043	.006
**Pubertal status**	-0.010	0.052	-0.199	.842	.001	-0.062	0.042	-1.478	.140	.004	-0.031	0.052	-0.591	.555	.001
**French-Canadian status** (ref: No)	-0.007	0.071	-0.099	.921	.000	-0.014	0.070	-0.201	.841	.001	0.058	0.065	0.889	.374	.001
**Smoking status** (ref: No)	-0.234	0.181	-1.294	.196	.003	-0.044	0.078	-0.563	.574	.001	-0.053	0.053	-0.992	.321	.002
**Household income**	0.000	0.001	0.138	.890	.000	-0.002	0.001	-1.498	.135	.004	0.000	0.001	-0.055	.956	.000
**Parental education**	-0.010	0.015	-0.668	.505	.001	0.000	0.015	-0.033	.974	.000	-0.014	0.015	-0.948	.344	.002
**Social support** (cumulative score)	0.002	0.374	0.004	.997	.000	-0.240	0.224	-1.073	.284	.002	-0.367	0.232	-1.580	.115	.004

## Discussion

This study aimed to test the relation between social support and low-grade chronic inflammation in a population-based sample of children and adolescents. As hypothesized, higher social support was significantly associated with lower CRP. The magnitude of the effect observed was comparable to previous research linking social support and inflammation in meta-analyses of the adult literature (*R*^2^ = .005) [17] and emerging findings in the child and adolescent literature [33–40, 43, 44]. While small, these generally consistent effect sizes imply a robust association between social support and inflammation across the lifespan. The small magnitude is not unexpected given that inflammation is only one of many pathways linking social support to health; the complex nature of inflammatory processes and their unmeasured biological influences encumber the interpretation of the effects. Further, the construct of social support is not harmonized in the field: heterogeneous measurement of different sources and types of support hinders meaningful synthesis, and in turn, obscures interpretation of findings linking inflammation and social support. Given the similar strength of association between this study and those within adults, these findings are promising for the pediatric literature. Future research should explore the relation between social support and inflammation across sex and age.

Among the emerging findings in children and adolescents, greater social support (feeling valued and cared for) has been previously linked to lower chronic inflammation. For example, higher parental support and positive behaviours have been related to lower CRP [33–36]. Inversely, negative aspects of social support (e.g., absence, poor quality, harsh climate) have been linked to higher chronic inflammation [37–40]. Results of the present study replicate these findings, and extend this work to a larger, population-representative sample across three age groups, and use a broader, more encompassing definition of social support. Conceptualizations of social support that include a wider network of home environment and neighborhood have shown that lower support (i.e., unsafe, fewer resources) are associated with higher levels of CRP in children and adolescents [43, 44]. Finally, prospective and retrospective evidence suggests the association between childhood social support and chronic inflammation tracks into adulthood [29–32]. The present findings indicate that the strength of the association emerges across age groups, with stronger associations observed among 13- and 16-year-olds. Altogether, findings suggest there is a social support gradient for inflammation across the lifespan, beginning in early adolescence.

Questions remain about the pathways linking social support and chronic inflammation. Sex has been posited as a moderator within social isolation and social integration models (few studies have considered gender; see below) [58]. Social isolation is thought to have a threshold effect: insufficient social support increases health risk via negative psychological states, poor self-care, and risky health practices, leading to increased inflammation. Inversely, social integration is thought to have a graded relation: having greater social support (e.g., number of social roles) decreases health risk via more adaptive cognitions, better emotional state, and healthier behaviours [58], leading to lower inflammation. For example, in a systematic review of social support and cardiovascular health risk in adults, sex moderated the association between social support and health risk; men had graded effects (i.e., social integration) while women had both graded and threshold effects (i.e., social isolation) [58]. In the present analysis, a graded association between social support and chronic inflammation was observed similarly in boys and girls. For example, increasing perceptions of greater support from anyone in the social network (e.g., family, peers, teachers, school, neighbourhood) in both boys and girls were linked to decreasing levels of chronic inflammation. Further, the high majority of boys and girls reported high levels of social support and social integration; only 4% endorsed social isolation (i.e., social support cumulative score below zero). Sex is commonly included as a covariate in childhood studies testing the association between social support and chronic inflammation; while no studies-to-date present sex-stratified results, mean differences have been reported. Notably, in the stratified analyses of the present study, the magnitude of the association between social support and inflammation was similar to that of the overall non-stratified analyses, but was no longer significant due to likely power limitations. Similar to past findings, girls reported higher social support [59–61] and had higher CRP [62], compared to boys. Mean level differences observed between boys and girls may be attributable to biological (sex) and/or social (gender) differences. Biologically, males and females have differing levels of pubertal timing, hormone production, adiposity, and fat mass distribution, which alter the CRP trajectory [63–66]. Socially, there is evidence that feminine individuals both seek and perceive higher levels of social support from their networks than masculine individuals [67]; though, few studies have considered gendered effects in relation to inflammation and health. Future research should consider how sex/gender and related variables (pubertal status, same/different-sex relationships) contribute to bidirectional models linking social support and chronic inflammation.

CRP levels in the present study were comparable to those previously reported in the pediatric literature (most < 3mg/L) [68]. Values of CRP that were below the detectable limit were imputed; this preferred approach to handle non-detects provides more accurate estimates and is deemed more robust against heteroscedasticity of errors [48]. CRP is low during adolescence and increases throughout adulthood [69]. The limited variability of CRP levels in this sample of youth (consistent with expected values), may have contributed to the null findings when stratified by age. In other words, when the CRP range is constrained, especially within 9-year-olds, there are power limitations to detect a significant association. Other inflammatory markers not assessed in this study (e.g., IL-4, IL-6, TNF-alpha) may be more prevalent at younger ages with lower fat mass and more sensitive to the developmental onset and progression of chronic inflammation. Furthermore, less invasive sampling methods (e.g., saliva) have been shown to have higher sensitivity to CRP and other inflammatory markers in adolescents [70]. Future research should assess additional early inflammatory markers and precursors as these may better reveal the emergence of the relation between social support and chronic inflammation early in the lifespan.

Covariates in this study included age, sex, BMI Z-score, medication use, puberty, ethnoracial status, smoking, household income, and parental education, all of which have been previously shown to be associated with inflammation. Unexpectedly, French-Canadian status, smoking status, household income and parental education were not associated with CRP in the present study. (Post-hoc exploratory analyses revealed the interactions of socioeconomic status with social support were also not significant.) The use of French-Canadian status as a proxy for race or ethnicity is a limitation of the dataset. It is plausible that other measures of ethnoracial status and/or culture may be related to social support and/or CRP. It is important for future research to examine the association between social support and chronic inflammation within a larger, cultural context. Additionally, prior researchers have also adjusted for physical activity, household smoking exposure, perceived and objective socioeconomic status, and stressor exposure (perceived or interpersonal) and their relation to CRP [71–73]. Future research should consider these covariates when testing the complex association between social support and inflammation and its myriad mediators and moderators. Higher order measurement of social support may help to delineate critical causal pathways and targetable predictors of chronic inflammation.

### Strengths, limitations, and future directions

The richness and quality of the QCAHS dataset, despite noted limitations, was an important strength of this study. First, this multi-stage sample survey of Quebec youth was a large, population-representative study that included information about children and adolescents’ social support combined with CRP values. Most studies conducted to date have only included small samples of youth (*N* < 200) that pose generalizability limitations. Second, it is rare to have fasting blood draw data for such a large number of participants, especially in a pediatric sample. As described earlier, the present sample included those who consented to blood draw and CRP assays. Compared to the entire QCAHS sample, this CRP subsample was slightly older and more advanced adrenarche development (puberty). However, these differences are likely not meaningful, as the subsample was only 3 months older and 0.08 stages more advanced. Third, this dataset presented a unique opportunity to examine the cross-sectional relation between social support and chronic inflammation with generalizability to the larger population of children and adolescents in Quebec. It is recognized that cross-sectional data precludes the examination of causal inferences (e.g., mediation, direction of association). However, the sampling design included three distinct age groups (9-, 13-, 16-year-olds), which provides valuable information spanning childhood and adolescent development. We recognize that additional work needs to be done to evaluate whether processes may differ across the lifespan. Fourth, the QCAHS was conducted in 1999, which could be deemed “old data”. While the data may no longer be population-representative (e.g., mean level of CRP may be higher given larger BMI trends over 2 decades), the *association* between social support and chronic inflammation would not necessarily be expected to change over time, which is the key focus of the present study. Ultimately, the QCAHS dataset provided an exceptional resource to examine the relation between social support and chronic inflammation in youth and to address important gaps in research within the extant literature.

The conceptualization and measurement of social support introduced strengths and weaknesses that merit consideration. The QCAHS survey was originally designed to examine social constructs and lifestyle behaviors linked to cardiovascular risk factors [46]. At the time of inception, the decision of which questionnaire items to include was guided by both the ability to make comparisons with other population-representative surveys at that time (e.g., Youth Smoking Survey [55]) and practical choices to minimize administration time, which is a common challenge for large epidemiological surveys. The secondary use of the QCAHS dataset precluded our ability to refine item wording or to use standardized measures of social support. We strived to optimize information available by creating a cumulative score for all items pertaining to social support. In fact, the aggregated score yielded remarkable psychometrics in the present study. Raters had excellent congruence in their selection of social support items, which included both source-specific and general questions about support in one’s social network. Further, the cumulative support score had good internal consistency. Nevertheless, the lack of a standardized measure of social support is a recognized limitation of the study. Most commonly used standardized social support measures for children and adolescents include the Student Social Support Scale (1999; 60 items) [74], the Child and Adolescent Social Support Survey (2002; 40 items) [75], the Social Support Scale for Children (2012; 24 items) [76], and the Social Support Questionnaire for Children (2016; 50 items) [77]. The majority of these scales have 40 to 60 items, require roughly 20 minutes for administration time, and therefore, few have been included in large population surveys (e.g, *N* > 1000). Further, these measures use a source-centric approach and categorize social support from parents, teachers, peers, and other sources. Obviously, these standardized measures were not used as they were developed and validated *after* the implementation of the QCAHS survey in 1999.

Curiously, while a source-centric approach is dominant in the child and adolescent social support literature, more nuanced subconstructs are used within the adult literature. For example, in adults, social support is largely defined as the *type* of support one receives: structural or functional [15]. Structural characteristics include the size of one’s social network and/or the degree of social integration one experiences (i.e., number of support sources in one’s network). Functional characteristics include the support processes that these networks serve, which can be further divided into two functional processes: *received* and *perceived* support [15]. Received support (i.e., what support one “gets”) captures interactions that are experienced such as help or advice during a crisis. Perceived support (i.e., support one “perceives” as available) captures beliefs of support availability that one can acquire from their network if necessary. To date, while the conceptualization of social support during childhood and adolescence has not been defined from these perspectives, closer inspection of standardized questionnaire items reveals that the assessment of functional support is embedded within the language of the item wording (e.g., makes me feel better, helps me solve problems). It is not yet clear whether there is a ubiquitous conceptualization of social support, or its subconstructs, that is contiguous across the lifespan. Another conceptualization used in the literature applies a more encompassing, macro perspective of broader social support. In fact, in the seminal meta-analysis by Holt-Lunstad and colleagues [9], broader (multi-dimensional) measures of social support yielded the strongest associations with longevity. Similarly, Kumar’s international study [10] demonstrated that a single question of broad social support (i.e., “If you were in trouble, do you have friends and relatives you can count on to help you whenever you need them, or not”) was significantly associated with self-reported health. A broader more encompassing conceptualization of social support, reflecting a possible higher order construct, may be more robustly associated with health than conceptual silos that parse social support narrowly by type or source. Nonetheless, parsing support by these narrow subconstructs can be useful to uncover mechanistic processes as exposures and processes at every level of one’s social ecosystem, from macro to micro, interact to influence health. Importantly, multiple perspectives of the conceptualization of social support are complementary and advantageous for the field.

Over the past two decades, children’s relationship and social support processes have evolved parallel to advances in technology and social media. In 1999, at the time of the QCAHS study, most social media platforms did not exist. In 2017, 76% of adolescents report using social media and over 90% use social media to connect with friends every day (e.g., over 1h per day) [78, 79, as cited in 78]. Since the onset of the COVID-19 pandemic, social media use has been the predominant means by which people connect with others and experience social support given social distancing mandates [80]. Social media introduces challenges to the conceptualization and measurement of social support. Social media may simply present an alternative *mode* to connect with loved ones (i.e., modes include in-person, letters, phone, video, social media platforms). On the other hand, it may create *new forms* of social support not redundant with existing support. For example, connectedness with one’s social media network (e.g., receiving anonymous “likes’’ or tweets from strangers) provides unique opportunities for experiencing social support. It is plausible to consider that social media use and/or deriving social support from social media groups may differ across marginalized populations and ethnoracial groups. There are emerging findings that social media may create unique and important opportunities for individuals from minority groups to feel more connected to others [81, 82]. Altogether, the measurement of social support, selection of suitable standardized measures, broader social support conceptualizations, and modern advances of social media introduced methodological considerations within the current study.

There are key recommendations to advance work on social support and inflammation in youth and to elucidate these findings. This study should be replicated in large samples, including population-representative cohorts. As the present findings were cross-sectional, they precluded the examination of a bidirectional association between social support and inflammation, and no causality can be inferred; future prospective studies would provide valuable data to investigate the causal nature of the relation and information about the predictive utility of social support. Moving beyond associations, it is valuable to investigate mechanisms (e.g., IL-6 production, stress buffering) and moderators (e.g., sex, gender) of the association between social support and inflammation to better understand the pathophysiological process. The construct and measurement of social support should continue to be carefully considered in future research. There is some evidence that a broader, more encompassing conceptualization using higher order macro-level items may provide a complementary perspective to existing conceptualizations of social support that are source-centric or isolate subconstructs. The importance of using psychometrically-sound, empirically-validated measures of social support remains fundamental for either conceptualization approach. Relatedly, it will be imperative that the role of social media and online platforms be evaluated within social support conceptualizations in future work.

## Conclusion

Social support predicts multiple health outcomes in adults, including those related to inflammation and immune functioning. There is less evidence for this relation in children and adolescents. In the present analyses, social support was associated with lower chronic inflammation across 9-, 13-, and 16-year olds. Remarkably, the magnitude of effect was similar to that previously observed among adults. The question remains how social support “gets under the skin” and leads to inflammation. Pathogenic mechanisms linking social support and CRP should be examined in future work. Pediatric studies offer a distinct advantage for investigating when and how chronic inflammation develops and its trajectory with social support across the lifespan. These findings contribute to the current state of knowledge about the relation between social support and inflammation and have implications for improving our understanding of the pathophysiology of systemic inflammation and susceptibility to disease.
